# Fgf8-Related Secondary Organizers Exert Different Polarizing Planar Instructions along the Mouse Anterior Neural Tube

**DOI:** 10.1371/journal.pone.0039977

**Published:** 2012-07-06

**Authors:** Ivan Crespo-Enriquez, Juha Partanen, Salvador Martinez, Diego Echevarria

**Affiliations:** 1 Instituto de Neurociencias de Alicante, Consejo Superior de Investigaciones Científicas–Universidad Miguel Hernandez, Alicante, Spain; 2 Department of Biosciences, Division of Genetics, University of Helsinki, Helsinki, Finland; Institut de la Vision, France

## Abstract

Early brain patterning depends on proper arrangement of positional information. This information is given by gradients of secreted signaling molecules (morphogens) detected by individual cells within the responding tissue, leading to specific fate decisions. Here we report that the morphogen FGF8 exerts initially a differential signal activity along the E9.5 mouse neural tube. We demonstrate that this polarizing activity codes by RAS-regulated ERK1/2 signaling and depends on the topographical location of the secondary organizers: the isthmic organizer (IsO) and the anterior neural ridge (anr) but not on zona limitans intrathalamica (zli). Our results suggest that *Sprouty2,* a negative modulator of RAS/ERK pathway, is important for regulating Fgf8 morphogenetic signal activity by controlling Fgf8-induced signaling pathways and positional information during early brain development.

## Introduction

Proper embryonic development requires an accurately orchestrated complex network of interactions between signaling and transcription factors. Secreted signaling molecules (morphogens) organize fields of surrounding cells into molecular patterns and are tightly associated to the concept of positional information. This concept implies that a cell reads its position and determines its developmental fate/response according to a concentration gradient of these extracellular factors [1]. These morphogens form long-range concentration gradients emanating from discrete sources and diffusing across the target fields [2]–[5].

The process of neurulation in vertebrates implies a major morphogenetic step for the initiation of brain regionalization. Localized signaling centers along the tube (called secondary organizers) and the morphogens emanating from them have a key role in refining the subdivisions of the embryonic brain. Among other morphogens, Fibroblast Growth Factors (FGFs) are a family of structurally related polypeptides with pleiotropic activities and are involved in a signaling system conserved from insects to humans [6]. Most FGFs mediate their biological responses as extracellular proteins by binding to and activating cell surface tyrosine kinase receptors (FGFRs). Three receptors, FgfR1, 2 and 3, are expressed in the vertebrate neural tube [7], [8], FgfR1 being the important for morphogenetic activity of FGF8. Out of the 22 known FGFS, FGF8 has been proven to be a crucial morphogen for early vertebrate brain patterning [9]–[12]. *Fgf8* is expressed preferentially at the so-called secondary organizers [13]–[16]. For more than a decade, the Isthmic organizer (IsO) has been used as a model to understand the morphogenetic activity of FGF8 and the planar induction mechanisms during mes- and rhombencephalon development in vertebrates [17]–[22].

Inactivation of *Fgf8* transcription at early neural plate stages causes death of the entire mesencephalic and cerebellar primordia revealing a requirement for FGF8 signal in survival of neural progenitors [22]. If FGF8 activity is only moderately reduced, the anterior midbrain appears normal, but posterior midbrain, isthmus and vermis are lost indicating concentration dependency of this signal activity [23], [24]. Moreover, misexpression of *Sprouty2* (one of the negative feedback modulators of FGF8 signaling; [25], [26]) moderately reduces FGF8 signaling in the IsO causing cell death in the anterior mesencephalon and rostralization of the remaining caudal midbrain epithelium suggesting that cell survival and patterning are independent properties [27].

Eight FGF8 isoforms have been identified so far, but only FGF8a and FGF8b isoforms have been related with IsO activity [28], [29]. They have different signaling activities over the neural tube depending on the signal concentration and receptor binging affinity [9], [29], [30]. Only a strong FGF signal mediated by FGF8b activates the Ras extracellular signal-regulated kinase (ERK) pathway, which is sufficient to induce cerebellar development [31]. In chick, ERK1/2 induction is afterwards downregulated by *Sprouty2* [32]. On the other hand, a lower level of signaling by FGF8a, FGF17 and FGF18 induces exclusively midbrain development [29], [31], [33]. Numerous feedback loops are known to maintain appropriate mesencephalon/cerebellum development and gene expression profiles around the IsO [19], [30], [34]. In fact, the duration of *Fgf8* expression in the IsO, and the strength of its signal activity seem to be crucial for the specification of these brain regions [35].

Three major intracellular signaling pathways can carry out the transduction of FGF signal during embryogenesis: PI3Kinase, PLC-gamma and Ras/MAPK (reviewed by [12], [36]). Phosphorylation of Extracellular signal Regulated Kinase 1/2 (ERK1/2) is a crucial step of the Ras-MAPK intracellular pathway. In early frog, fish, chick and mouse embryos, ERK1/2 activity depends on FGF signaling making the detection of di-phosphorylated forms of ERK1 and ERK2 (dpERK) useful readouts of FGF activity. In vertebrate embryos, ERK1/2 phosphorylation pattern profile is discrete, dynamic and it largely correlates and with *Fgf8* gene expression domains [24], [37]–[39].

The proposed mechanism by which the signaling of FGF8 spreads over a field of target cells in zebrafish is established and maintained by two essential factors: firstly, free diffusion of single FGF8 molecules away from the secretion source through the extracellular space and secondly, a sink function of the receiving cells regulated by receptor-mediated endocytosis [40], [41]. However, the precise shape of the FGF8 morphogenetic activity is still unclear during the early mammalian brain regionalization. It is also important to understand how the FGF8 signaling expands from the IsO in order to be interpreted as positional information by the nearby neuroepithelial cells.

Here, we address these questions using the mouse IsO as experimental model system. The study discloses position related preferences of neuroepithelial cells to FGF8 planar signal activity. This differential orientation and polarity of the FGF8 signal is directly dependent on the spatial position of mouse Fgf8-related secondary organizers and on the activity of a negative modulator Sprouty2. Our findings reaffirm the existence of positional information encoded by the FGF8 morphogenetic activity in neuroepithelial cells along the vertebrate neural tube.

## Results

### ERK1/2 Phosphorylation Reveals the Longest-range Form of FGF8 Morphogenetic Activity From the Mouse IsO

**Figure 1:**
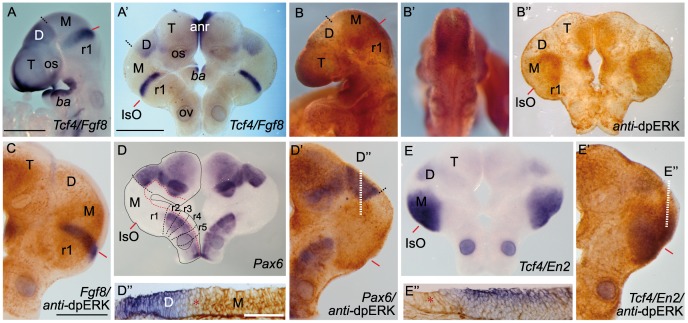
Phosphorylation patterns of ERK1/2 (dpERK) enzymes in the anterior neural tube. Whole mount in situ hybridization (ISH) of E9.5 mouse embryo (A,B,B’) and corresponding organotypic neural tissue cultures of mouse E9.5 anterior neural tube (A’,B”,C-E”; ONTCs; [42]) where it shows the maintenance of gene expression profiles such us *Fgf8*, *Tcf4* (A,A’) (used to delimit main brain subdivisions such the diencephalon (D; *Tcf4* positive) and the mesencephalic anlage (M; negative staining), *Pax6* D-D” (delimiting di-mesencephalic boundary and rhombomere 1–2 limits) and *En2* E-E”. In B-B”) are photomicrographs of E9.5 mouse embryo with *anti-*dpERK Immunohistochemistry (IHC) taken from lateral (B) and caudal (B’) sides and the corresponding IHC in ONTCs. (C) double staining procedure: ISH (in blue) for *Fgf8* and IHC for dpERK (dark brown) to localize inside the dpERK domain the position of the IsO, marked by the solid red line. (D-E”) photomicrographs of same ONTCs in which first a whole mount ISH for *Pax6* (D) *or Tcf4/En2* (E) were made and afterwards IHC against dpERK. (D’,E’ respectively). Dashed lines mark the main transversal (in black) and longitudinal (in red) brain subdivisions. These ONTCs were cut into transversal sections to the isthmic constriction (D”,E”) to proof that indeed dpERK expression reaches diencephalic anlage (D”; see asterisk; rostral is left) and has a wider expansion than *En2* expression (E”; see asterisk; rostral is left). anr is anterior neural ridge secondary organizer; ba is branchial arch; IsO is isthmic organizer; os is optic stalk; ov is otic vesicle; r is rhombomere; T is telencephalon, D, diencephalon, M, mesencephalon. Scale bars are 0,5 mm except in D”, E” they are 100 µm.

We first compared the distribution of phosphorylated forms of ERK1/2 activity (Figure 1B ^s^; [38]) to the expression pattern of *Fgf8* (Figure 1A,A”) and the main *Fgf8* downstream genes (Figure 1C-E”) in E9.5 wild-type mouse embryos and organotypic cultures of neural tube explants (ONTCs; [42]). In both models, we also corroborated the expression patterns of the *Fgf8* downstream negative modulators *Sprouty2*, *Sef*, *Mkp3* showing their gradient distribution being strong near the FGF8-related secondary organizers (Figure S1 and [25], [43], [44]).

In E9.5 whole mount embryos, immunodetection of ERK1/2 did not show the same distribution as the FGF8 modulators (Figure 1B,B’). In fact, when using E9.5 ONTCs the immunostaining against phosphorylated forms of ERK1/2 showed an almost non-gradient pattern, facing now the ventricular side of the IsO territory (Figure 1B”,C; [42]). Moreover, in E9.5 ONTCs ERK1/2 phosphorylation was detected over almost the entire mesencephalon (also positive for *Meis2* transcription factor; see Figure S1A) and the entire rhombomere 1 (r1; positive for *En2* and limited caudally by *Pax6*; Figure 1D’,D”). Rostrally, ERK1/2 activity staining reached the ventral parts of the mesencephalic-diencephalic boundary (based on the caudal gene expression limit of either *Pax6* or *Tcf4*; n = 28/33; Figure 1D’-E”) leaving a mesencephalic alar plate wedge domain free of expression. Caudally, the immunodetection adjoined to the expression of *Pax6* at rhombomere 2. Therefore, at this developmental stage phosphorylated forms of ERK1/2 appeared to be the longest-range marker for FGF8 activity.

**Figure 2:**
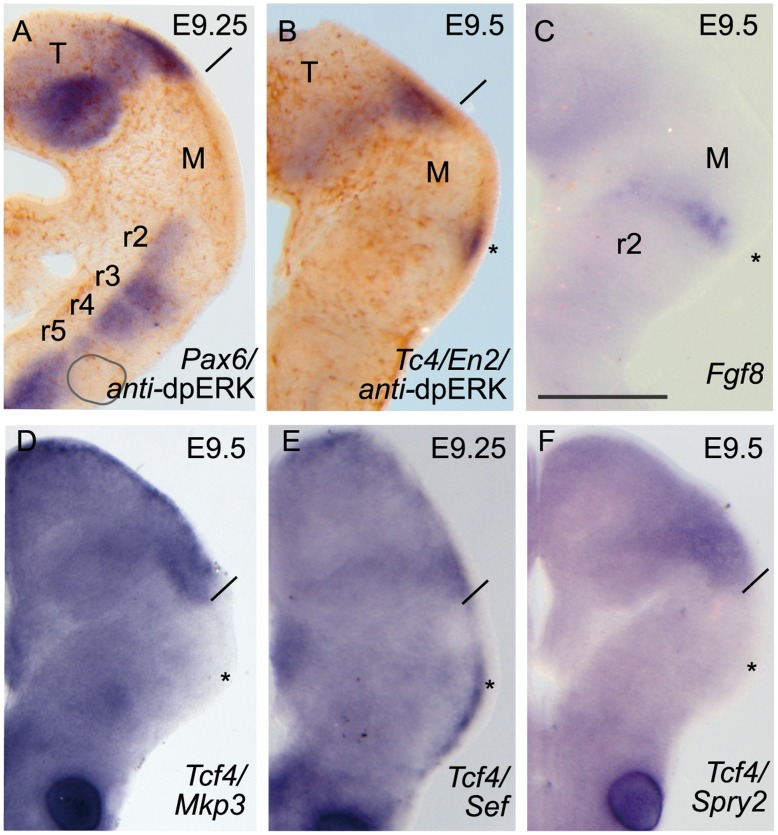
Low threshold of FGF8 protein levels disrupts ERK1/2 phosphorylation patterns. .DpERK immunodetection was absent in the isthmic domain using ONTCs severe hypomorphic mouse mutant (A, B; *Fgf8 ^neo/null^* [45]). Yet a small tip of *En2* (B) positive expression was visible at the most dorsal parts, probably by the maintenance of *Fgf8* (C) expression. Under these mutant conditions, none of the FGF8 signal negative modulators *Mkp3* (D), *Sef* (E) *Sprouty2* (F) were observed at IsO. Asterisks indicate the position of abolished isthmic region and solid line the boundary between diencephalon/mesencephalon. Scale bar in C is 0,5 mm for all images.

To demonstrate that ERK1/2 phosphorylation was controlled by FGF8 activity on these territories at E9.5, we used mutant mice with reduced levels of FGF8 (*Fgf8* hypomorphs; [23], [45]). Immunostaining of dpERK at mid- and hindbrain regions was completely absent in these mutant mice (n = 7/7; Figure 2A, 2B). This absence was concomitant with downregulation of *Fgf8* expression and of FGF8 downstream negative modulators genes such as *Sef*, *Mkp3* and *Sprouty2* (n = 6/6; Figure 2C-F; for comparison see Figure S1). In the *Fgf8* hypomorphs *Tcf4* expression pattern (used as a landmark for caudal diencephalic limit) did not change. Surprisingly, at this developmental stage we still found a small portion at the most dorsal area of the isthmic constriction where *Fgf8*, *Sef* and *En2* transcripts were expressed (asterisks in Figure 2B,C,E). However, this reduced expression was not enough to maintain full IsO morphogenetic activity [23]. Therefore, ERK1/2 phosphorylation at mouse midbrain and rostral hindbrain seems to be strongly linked to the FGF8 signaling activity coming from the IsO.

### FGF8 Signaling Activity Exerts Different Tissue Preferences Along the Anterior Posterior Neural Tube Axis

**Figure 3:**
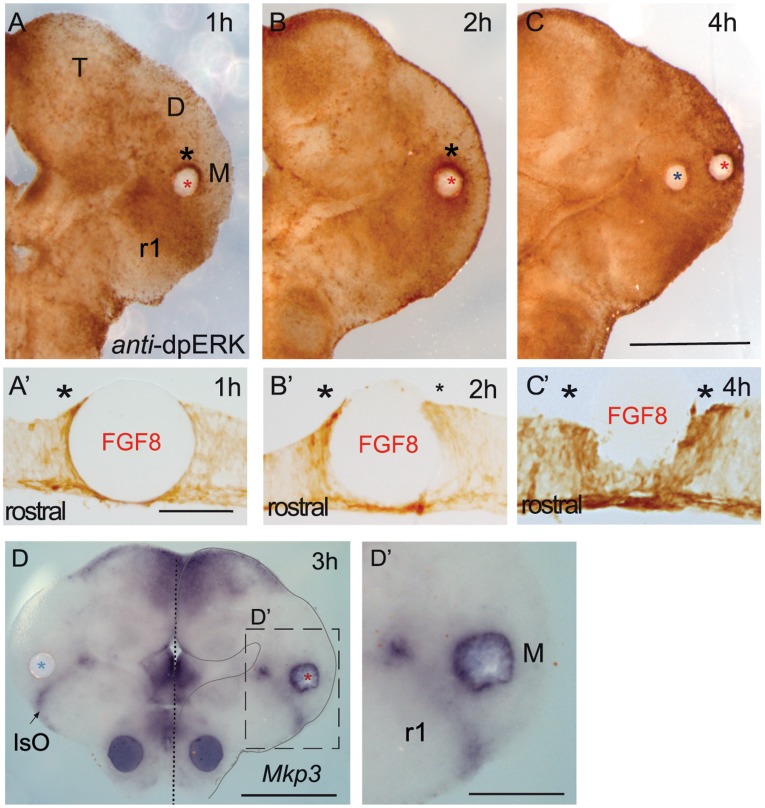
FGF8 planar induction from IsO has initial tissue preferences in mesencephalon. Classical FGF8 soaked bead implantation in mesencephalon induces ERK1/2 activity before earliest induction of mRNA (*Mkp3*) could be detected (D). Interestingly asymmetric distribution is detected during the first two hours after incubation (A, A’ and B, B’). This asymmetry is lost from 3 hours onwards after bead implantation (C, C’). In cryostat sections a mesencephalic bead implantation induced highly intense ERK1/2 activation as observed at the rostral side of the bead after 1 hour of incubation (A’). After 2 hours, ERK phosphorylation is detected caudal to the bead (B’; see small asterisk). Finally ERK activity is homogeneously distributed at both rostral and caudal cells after 4 hours (C’). Red asterisks indicate an FGF8 soaked bead; blue asterisk indicate a PBS bead. Scale bars are 0,5 mm except for A’, B’, C’ that are 50 µm, in D’ is 0,25 mm.

**Figure 4:**
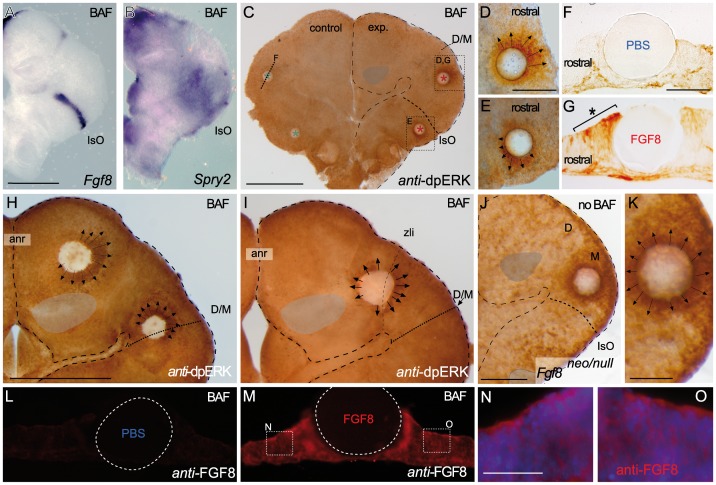
Bafilomycin A1 (BAF) treatment demonstrates the polarization of ERK activity by FGF8 signal activity along the neural tube. A-C) BAF amplifies ectopic ERK1/2 activity-related FGF8 induction revealing a clear polarized distribution of ERK1/2 activity around FGF8 soaked beads depending of the implanted bead; rostral to IsO (C,D,G), or caudal to IsO (C,E). Nonetheles, isthmic organizer morphogenetic activity seems unaffected for *Fgf8* (A) and negative regulator *Sprouty 2* (B) expressions. Note that PBS bead implantation in control side (blue asterisk in C and F) did not show any ectopic induction. Two hours after bead implantation a clear amplified and almost non-homogeneous ERK1/2 activity was detected rostrally in the mesencephalon (rostral to the IsO), which was detected caudally when bead was placed in hindbrain (caudal to the IsO) territories (E). In telencephalic vesicles, caudal to the anr (H) the polarity of ERK activation was reversed. This polarized dpERK detection around the bead is lost at the zli (zona limitans intrathalamica) region (I). Similar symmetric ERK-related FGF8 signal found in zli was seen when placing a FGF8 bead in the midbrain of *Fgf8* hypomorphic mice (J,K). Importantly FGF8b protein distribution (M) was observed apparently in equal intensity and range at rostral (N) and caudal (O) sides of the bead (for comparison with PBS bead in panel L). Scale bars are 0,5 mm in A, B, C, H, I, 200 µm in D, E, J, 100 µm in F, G, K, L, M, and 50 µm in N, O.

We next characterized the molecular dynamics of FGF8 signaling activity coming from the IsO, analyzing ERK1/2 activity after ectopic implantation of FGF8 sources (Figure 3 and 4; see material and methods). Ectopic induction of *Mkp3* was the first transcript detected only after 3 hours of FGF8b soaked bead implantation to the mesencephalon (Figure 3D,D’ and [46]). On the other hand, ectopic ERK1/2 activity was detected already before one hour of incubation with FGF8b beads (Figure 3A). Interestingly, the ERK1/2 phosphorylation staining was distributed asymmetrically around the bead in the mesencephalon (Figure 3A,A’). High intensity of staining was detected only at the rostral side of the bead (n = 9/13). With longer incubation time periods (2 hours; Figure 3B,B’), ectopic dpERK immunostaining started to be detected also caudal to the bead but was still induced higher in rostral cells. Only after 3 to 4 hours of FGF8b bead incubation, ERK1/2 activity was detected symmetrically around the bead (Figure 3C,C’). In all cases, control PBS soaked beads implanted on the same neuroepithelial positions and same time periods neither showed induction of ERK1/2 activity nor molecular induction of *Fgf8* downstream genes (blue asterisk in Figure 3C,D). This early asymmetric phosphorylation of ERK1/2 raised the possibility that FGF8 morphogenetic activity may confer positional information to the neural tube encoded already at the intracellular signaling pathway level along its anterior-posterior axis.

To further investigate the causal mechanisms of this unbalanced distribution of ERK1/2 activity at early steps of FGF8 signaling, we searched for amplification of the intracellular ERK1/2 activity. Recent work has proposed as an explanation for the establishment of FGF8 morphogen gradients by endocytosis and degradation of the Fgf8 protein [41]. Therefore, we decided to pharmacologically block the lysosomal pathway to prevent FGF8 degradation after endocytosis. Using Bafilomycin A1 compound (BAF; see material and methods) endocytosed FGF8 should maintain within the endosomes and still trigger ERK1/2 activity [47]; while the extracellular FGF8 protein should continue to be taken up by the cells. After 2 hours of 1 µM BAF treatment [48] E9.5 ONTCs still maintained similar molecular IsO activity and gene expression patterns to those observed in controls (Figure 4A,B). When we implanted FGF8b soaked beads during the BAF treatment (2 hours) a significant amplification of ERK1/2 phosphorylation signal occurred (compared Figure 4G with non-treated explants bead implantation assays 3B’). PBS-beads did not produced and ERK1/2 ectopic induction (blue asterisks in Figure 4C,F). In addition, this treatment disclosed an intensification of the polarization effect (Figure 4C-I). The asymmetric distribution of ERK1/2 immunodetection around the bead was clearly localized and extended rostrally when those beads were implanted in the mesencephalon (n = 17/20; Figure 4C,D,G). Only when FGF8b beads were placed on the rhombencephalon a reversed polarization distribution of phosphorylated ERK1/2 staining was detected (in these cases caudal to the bead; n = 4/5; Figure 4C,E). FGF8b beads implanted ectopically at more anterior territories in caudal diencephalic anlage showed a less evident “crescent moon”-like ERK1/2 phosphorylation staining, with decreased expansion rostral to the bead (n = 5/5; Figure 4H). In fact, when these beads were placed at the thalamic/prethalamic boundary (n = 4/4; Figure 4I), the zona limitans intrathalamica (zli; [16], [49], [50]), they induced ERK1/2 equally distributed around the bead (symmetrical). Furthermore, when FGF8b beads were implanted in telencephalic regions, close to the mouse anterior neural ridge secondary organizer (anr; [13]) ERK1/2 activity was mainly induced in cells caudal to the beads (n = 7/9; Figure 4H). Therefore, FGF8b signal exerts differential responses on the neuroepithelium, which are encoded already at the level of the Ras-MAPK intracellular cascade activation along the anterior-posterior axis of the mouse neural tube (see below).

In order to exclude any differential spatial protein release from the FGF8b beads towards rostral or caudal directions, we analyzed distribution of the ectopic FGF8b protein in the neuroepithelial cells using a specific monoclonal antibody (see materials and methods). The results demonstrated an equal distribution of the protein at both caudal and rostral sides of the FGF8 beads in the ONTC neuroepithelium (n = 4/4; Figure 4N-4O). Finally, we checked also if the endogenous FGF8 activity levels contributed to the observed ERK activity asymmetries. Thus, we implanted FGF8b soaked beads into ONTC mesencephalon made from embryos homozygous for severely hypomorphic Fgf8 alleles [23]. In these mutants, the ectopic ERK1/2 phosphorylation was always induced symmetrically after 1 hour of bead incubation (n = 10/11; Figure 4J, 4K). Thus, the asymmetric effects of FGF8b beads on ERK1/2 phosphorylation seem dependent on proper FGF8 function, likely coming from the FGF8-related secondary organizers [12], [16].

### Topography of Secondary Organizers Determine the Polarization of FGF8 Signaling Activity Along the Neural tube Through Negative Feed Back Modulators

**Figure 5:**
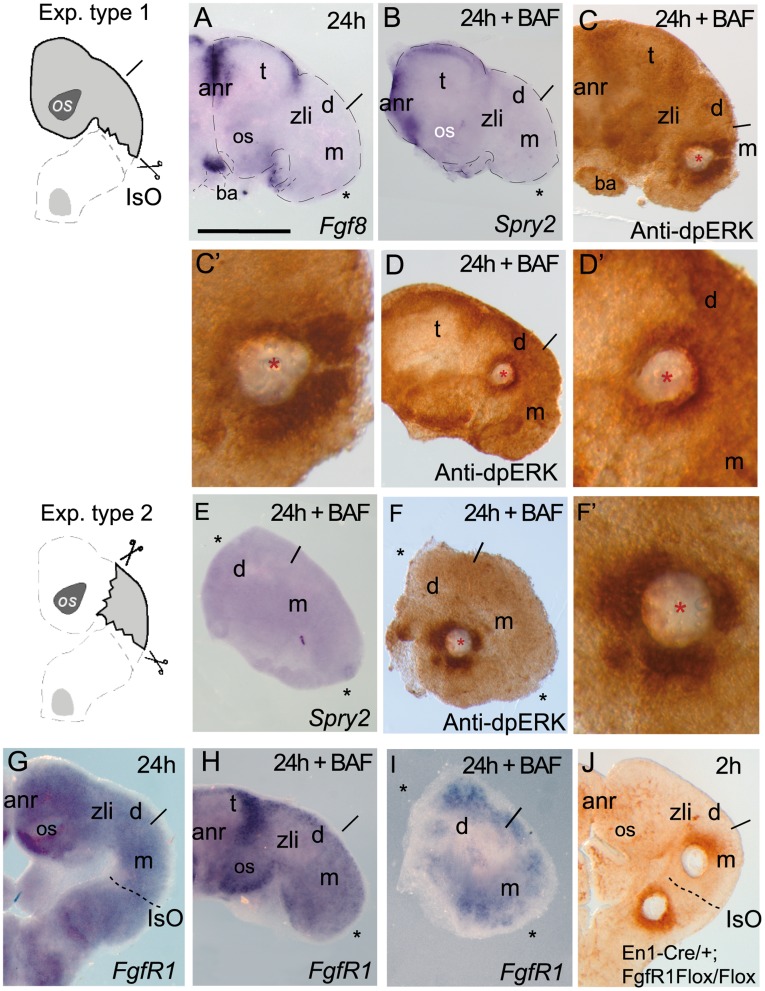
The position of FGF8- related secondary organizers determines the polarity of ERK1/2 activation. .A-D’) show the type 1 experimental manipulation in ONTCs where a dissection of the IsO region was made, left for 24 hours *in vitro* and thereafter it was incubated with BAF for 2h after an implantation of a FGF8b bead in mesencephalon (C-C’) or middle diencephalon (D-D’). *Fgf8* mRNA (A) and *Sprouty 2* (B) were maintained at anterior neural ridge (ANR), optic stalk (os) and branquial arches (ba) but they were absent in caudal regions of the ablated ONTCs. Bead implantations in mesencephalon modified ERK1/2 polarization towards caudal parts of the bead (C, C’; for comparison see Figure 4C). Bead implantations in the diencephalon maintained symmetric distribution of ERK1/2 activity around the bead (D,D’). In these experiments *FgfR1* expression (G) was maintained in IsO ablations (H). E,F,F” and I show type 2 experimental manipulation assays in ONTCs where rostral forebrain (anr included) and hindbrain (type 1 experiment) were ablated. Under these conditions and following the BAF incubation protocol, the tissue left did not express any FGF8 downstream genes (*Sprouty2*; D) and the ectopic induction of ERK1/2 activity was found symmetrically distributed around the bead (F and F’) on *FgfR1* positive domain (I). In fact the lack of *FgfR1* in the midbrain and hindbrain region, does not disturb ERK1/2 polarizing effects on both brain regions (J). Scale bars are 0,5 mm except for C’, E’ that are 100 µm.

To prove that FGF8-related brain secondary organizers were the sources for early ERK activity polarity, we conducted neural tissue ablation assays of these morphogenetic brain areas on E9.5 mouse ONTCs (see Figure 5 drawings). In type 1 assay the IsO was ablated and the remaining tissue was incubated for 24 h before BAF treatment or implantation of FGF8b soaked beads (Figure 5A-5D’, 5H). Under these conditions *Fgf8* transcripts (n = 12/15) and negative modulators of Fgf8 signaling such *Mkp3* (n = 4/4) and *Sprouty2* were not longer expressed at caudal mesencephalon (n = 5/5; Figure 5A, 5B). Meanwhile, *Fgf8* expression was still maintained at the anr and at the upper mesenchymal branchial arches (ba). In these ablation assays the main signaling receptor for FGF8, FGF receptor 1 (*FgfR1*; Figure 5G, 5H); [33], [51], [52]) was also maintained uniformly at the mesencephalon. Here, after 2 hours of FGF8b bead implantation in the midbrain and BAF treatment, we detected effects opposite to the previously observed polarized response of ERK1/2 in this region. Immunodetection of dpERK appeared now stronger at the caudal parts of the FGF8b bead than at rostral one (n = 11/14; Figure 5C, 5C’; compare with Figure 4C,H). Interestingly, FGF8b beads implanted at the central diencephalon (putative zli; Figure 5D, 5D’) still maintained the symmetrical distribution of ERK1/2 activity around the bead. Thus, these results support the idea that at E9.5 the murine isthmic organizer region must be the source for the initial polarizing cue on ERK1/2 activity related to FGF8b signal in the entire mesencephalon, caudal diencephalon and most probably rhombomere 1.

In type 2 assays the IsO and anr were ablated. Using the same conditions as type 1 experimental assays, the remaining neural tissue (mostly mesencephalic and diencephalic regions) showed no expression of *Fgf8* (n = 6/6) or *Fgf8* negative feedback modulators *Mkp3* (n = 7/7) or *Sprouty2* (n = 3/3) before any treatment (Figure 5E). The ablated tissue still showed traces of *FgfR1* transcripts in the mesencephalic territory (Figure 5I). After BAF treatment and FGF8b bead implantation into the rostral mesencephalon, phosphorylated ERK1/2 staining was observed symmetrically around the bead (n = 14/16; Figure 5F,F’). The staining was very similar to the results using ONTCs of *Fgf8* hypomorphic embryos or when the FGF8b beads were placed in the zli of wild-type ONTCs.

Finally, as an attempt to exclude any sensitive receptor mechanism underlying this initial polarizing activity exerted by FGF8b signaling we conducted FGF8b bead implantation assays on ONTCs of mutant embryos where FgfR1 was conditionally inactivated in the midbrain-rhombomere 1 region (En1^Cre/+^; FgfR1 ^flox/flox^; [51], [52]). These mutant ONTCs (n = 6/6; Figure 5J) disclosed same polarized ERK1/2 activity as when FGF8b beads were implanted on rostral and caudal sides of the IsO in wt ONTCs (see Figure 4). Importantly, in these experiments we did not use Bafilomycin A1 compound to arrest late endosomal pathway. Here, ERK1/2 activity immunodetection was more expanded on the neuroepithelium than in normal ONTCs suggesting a differential endosomal sorting and a high rate recycling characteristics of the FGFR1-FGF8b endocyted complex. Therefore, it seems that along the anterior neural tube, the gradient activity of FGF8b may result from different planar instructions regulated by the FGF8 feedback negative modulators.

**Figure 6:**
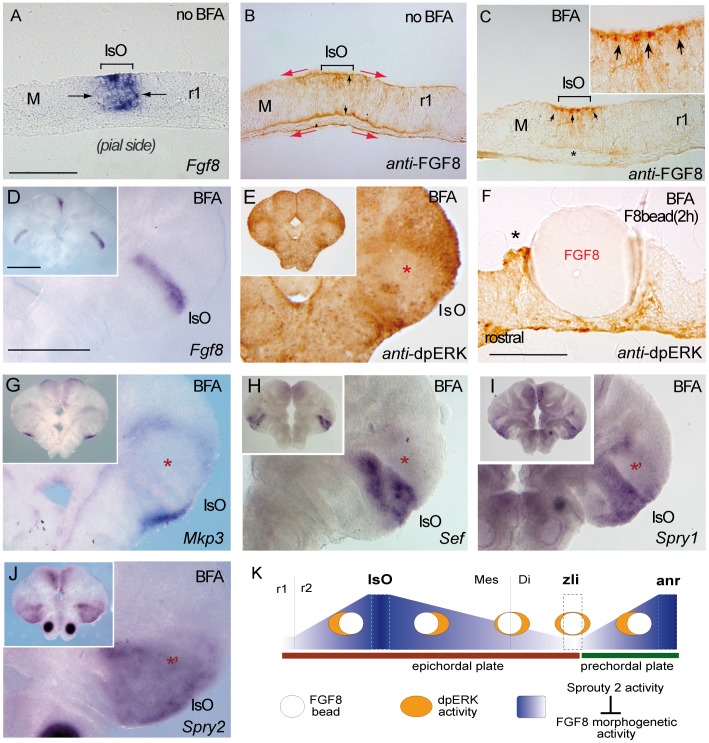
Brefeldin A (BFA) treatment inhibits ERK1/2 activity and modulates differentially Fgf8 negative feed-back regulators. A, B and C) are 12 µM cryostat transversal sections of mouse ONTCs to the isthmic constriction. A and B) are control example before BFA administration to the culture medium showing an ISH for *Fgf8* (A) and an Immunostaining for anti-FGF8 (B). Note the different domains of expression of the transcript (delineated by the solid line and arrows) and of the protein FGF8 (delineated by the red arrows). Note also that FGF8 immunodetection is detected both at basal and ventricular sides of the ONTCs (see black arrows in B). After 4 hours of BFA incubation (C-J) the mRNA of *Fgf8* was maintained at the IsO (D) while the FGF8 protein profile changed dramatically being accumulated only at the ventricular side (D) as small vesicle-like, (see arrows in the figure C and the magnified insert). Moreover, ERK1/2 activity disappears in Isthmic cells and nearby cells (E). Inside this negative gap, FGF8b beads still exerts polarizing ERK1/2 effects (F). Also inside this gap, genes such us *Mkp3* (G) and *Sef* (H) disappear in the mesencephalon while *Sprouty* family genes are maintained (I,J). K) represents the experiments and model by which FGF8 planar induction activity coming from mouse FGF8-related secondary organizers (IsO and anr) exerts a different tissue preferential signaling effects (based on the activation of ERK1/2). The direction of polarized ERK1/2 activity depends on the location of FGF8-related secondary organizers and the establishment of this positional information signaling is dependent mainly on FGF8 negative modulator system, particularly *Sprouty2* (blue gradient). Moreover, FGF8 morphogenetic planar instruction signals coming from rostral (anr) and caudal (IsO) diminish and loose their polarization effect at the diencephalic region (zli) resulting in an equilibrium state. Scale bars is 100 µm except for D,E, G-J which is 0,5 mm.

We therefore analyzed the contribution of the FGF8 feedback modulators (*Mkp3*, *Sef*, *Sprouty1/2*) during these initial planar instructions of FGF8 signaling. We decided to deprive pharmacologically the E9.5 mouse neural tube from any endogenous secreted molecule to the extracellular space (including FGF8 protein). Brefeldin A (BFA) was chosen for the ability to inhibit protein secretion in mammalian and other eukaryotic cells by interfering with the function of the Golgi apparatus, resulting in dysregulation of membrane traffic [53]. Before that, we detected the endogenous FGF8 protein distribution *in vivo* by Immunohistochemistry in whole-mount E9.5 mice with specific antibody against FGF8. The results revealed FGF8 staining either at the neuroepithelial intracellular or at extracellular levels in cryostat sections transverse to the IsO constriction (Figure S2A,B and [54]). FGF8 positive immunostaining was detected outside the limits of its mRNA expression, both caudal and rostral to the IsO. Moreover, the FGF8 protein was detected at both ventricular and pial sides of this pseudostratified neuroepithelium suggesting diffusion facilitation through basal lamina (Figure S2C,D) and [54]). Similar patterns of *Fgf8* gene expression and FGF8 protein distribution were also observed in cryostat sections of wild-type ONTCs (n = 3/3; Figure 6A,B). We then treated the ONTCs with BFA at 25 µg/ml (Figure 6C-J; [55]) for a period of 4 hours of incubation. Under BFA treatment, a positive anti-FGF8b immunolabeling was detected exclusively in the territory of the IsO and now restricted to the *Fgf8* gene expression domain (n = 3/3; compare Figure 6A, C). Interestingly, BFA treatment revealed a more intense immunopositive reaction for FGF8 only at the ventricular surface of the pseudostratified isthmic neuroepithelium (absent in the basal lamina; asterisk in Figure 6C). Also, the FGF8 immunostaining was detected as bead-like structures, resembling exocytic vesicles and interestingly only concentrated close to the ventricular surface (Figure 6C insert). These data strongly suggest that FGF8-producing cells (*Fgf8* mRNA positive) in the mouse IsO, secrete the morphogen near the lumen of the neural tube but the gradient of secreted Fgf8 protein forms a gradient along the basement membrane.

**Figure 7:**
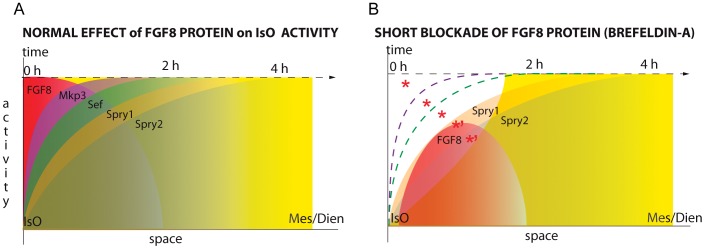
Initial FGF8 planar instruction effects in IsO after a short signaling deprivation. .Schematic representation of isthmic FGF8 feedback modulator genes regulation given by the isthmic gradiental FGF8 morphogen (and signaling) on the mouse mesencephalon. The graphic describes the distribution of dosage (activity) versus time and space of FGF8 and of *Fgf8* negative signal modulators. A) represents the normal FGF8 signal activity in the IsO (high levels of FGF8 protein; red solid wedge), during which FGF8 maintains at dose-dependent manner the different FGF negative modulators expression profile starting from *Mkp3* (purple solid curve), *Sef* (green) and finishing *Sprouty* genes (where low levels of FGF8 protein). The yellow background represents ERK activity. B) describes the presumed situation during Brefeldin a (BFA) treatment (4 hours) on the FGF8 morphogenetic activity. Thus, at isthmus the FGF8 protein level (a therefore morphogenetic activity: red solid bell-shaped curve) would be cero but some residual protein away from the source would still activate ERK (yellow solid slope curve). Inside this negative gap of ERK1/2 activity the expression of *Mkp3* (purple dashed curve line) and *Sef* (green dashed curved line) at the mesencephalon is completely absent. Nonetheless the residual FGF8 morphogen apart from the isthmus is enough to maintain *Sprouty1/2* expression in the mesencephalon in the absence of ERK activity (red asterisks’; see also [32], [76]).

More importantly, blockade of exocytosis caused a fast downregulation of ERK1/2 activity in the IsO domain (n = 4/5; Figures 6E and Figure 7A,B), while in territories further away, cells still remained dpERK positive. Thus, the sudden blockade of exocytosis of FGF8 protein secretion affected first the FGF8b producing cells (i.e. the isthmic organizers cell population) and gradually the neighboring territories away from them (see Figure 6E and Figure 7A,B). These results provide new cellular mechanistic information about the induction characteristics of the isthmic FGF8-expressing cells (see discussion). Therefore, these neuroepithelial cells required secretion of FGF8 to exert proper activation of FGF8 intracellular pathways. Interestingly, implantation of an ectopic FGF8b bead inside this negative phosphorylated ERK1/2 domain for (n = 4/5; Figure 6F) still exerted an asymmetric distribution of dpERK labeling after 2 hours of incubation (n = 2/2; Figure 6G). During this process of depriving the neural tube from extracellular FGF8 protein, *Mkp3* was not detected inside the negative phosphorylated ERK1/2 domain (n = 3/4; Figure 6H and Figure 7B). Of the other known FGF8 modulators [12] we found that *Sef* expression was completely downregulated in the mesencephalon but not in the r1 ((n = 3/3; Figure 6I). Moreover, the *Sprouty1* gene showed a similar pattern as *Sef* but with a less severe phenotype in the mesencephalon (n = 4/6; Figure 6J and Figure 7B). Finally, expression pattern of *Sprouty2* was maintained both in mesencephalic and rhombencephalic territories as in control ONTCs (n = 4/4; Figure 6K, Figure 7B and Figure S1B’). In conclusion, the specification of early FGF8-related positional information signaling that will induce later differentially biological responses at different distances from the IsO seems to be controlled by graded expression of the FGF8 negative modulators. Our results strongly suggest requirement for mainly *Sprouty1/*2, but not of *Mkp3* and most probably not of *Sef*, for the initial polarized response to FGF8 in the mouse midbrain.

## Discussion

The reliable mapping of active ERK signaling domains in Drosophila [56] and diverse vertebrates such as xenopus, zebrafish, mouse and chick [37]–[39], [57] have been obtained using antibodies specific to the di-phosphorylated forms of ERK1 and ERK2. We have demonstrated a close relationship between FGF8 signal activity coming from the isthmic region and ERK1/2 phosphorylation, using open-book E9.5 mouse neural tube organotypic tissue explants cultures (ONTCs; [42]), which also corroborated other *in vivo* studies [38], [39]. Investigators working in other vertebrate brain regions such as mouse telencephalon [58], [59], chick caudal hindbrain and spinal cord [60], [61] have proposed also ERK1/2 phosphorylation immunostaining as a direct readout tool of FGF signal activity. Although other pathways, such as integrins, cytokines and G-protein-coupled-receptors, can also activate the RAS-MAP-ERK pathway [62], the majority of ERK1/2 activity domains correspond to FGF signaling domains [24], [32], [38], [39], [63]. Nevertheless, the isthmic region remains the most reliable and sensitive model system for understanding FGF8 function in neural tube development in vertebrates [23], [41]. Here, we demonstrated that expression of neither negative feedback modulators of FGF8 signaling or phosphorylated forms of ERK1/2 were detected in the mid-hindbrain territories at E9.5 on *Fgf8* hypomorph mutant mice [23], [45] corroborating the tight close link between the morphogen FGF8 and ERK1/2 activity.

FGF8 downstream negative modulators have been used as indirect markers for the study of FGF8 signal activity in the vertebrate IsO [12], [16], [22]. These genes are expressed in same regions as *Fgf8* but in a wider and graded long-range pattern. In contrast, at E9.5, phosphorylated forms of ERK1/2 showed no clear graded patterns. In fact, the more homogeneous distribution of ERK1/2 labeling in the mid- hindbrain territories reached rostrally the diencephalic/mesencephalic boundary and caudally the rhombomere R1/R2 limit, unveiling the maximal long-range activity of endogenous FGF8 at this developmental stage. Other mouse IsO-related genes can reach similar neuroepithelial boundary limits before E9.5 such as *Engrailed1/2*, *Pax2* and *Sprouty1/2* but from E9.0 they become restricted closer to the isthmus [25], [43], [64], [65]. Moreover, we have found that ERK1/2 activity is also the fastest readout of FGF8b morphogenetic activity in the mouse anterior neural tube. Already at 60 minutes, FGF8b soaked bead implantations in the mesencephalon of E9.5 mouse ONTCs caused ectopic ERK1/2 activation. That makes the detection of ERK1/2 phosphorylation form a convenient tool for understanding early FGF8b morphogenetic signal interpretation [38], [39]. Similar experimental assays have been also described in the chick spinal cord at earlier stages of development [60].

The ability of a rapidly internalized receptor to signal after endocytosis is important to ensure the sufficient duration and intensity of signaling. However, this capacity requires receptors to remain active in endosomes and therefore able to di-phosphorylate ERK [47]. Several Receptor Tyrosine Kinases (RTKs), for example EGFR, remain ligand bound, phosphorylated and active in endosomes until late stages of endosomal trafficking, including the presence of a MAPK scaffold complex (reviewed in [47]). Following the recent findings on the endocytotic mechanism for Fgf8 morphogen in zebrafish IsO [41], we used Bafilomicin A1 (BAF), a highly specific inhibitor of vacuolar type H^+^-ATPase (V-ATPase; [66]) to amplify the ERK1/2 phosphorylation in cells induced by FGF8b signaling. Under these conditions we disclosed an asymmetrical distribution of ERK1/2 activity, but not of the FGF8 protein itself, around the FGF8 releasing bead. This differential planar tissue behavior in the ERK intracellular response depended on the position of the FGF8b bead relative to anterior neural ridge (anr) and isthmic organizer (IsO) (the FGF8-related secondary organizers; Figure 6K; [13], [19]). Importantly, the diencephalon behaved very differently from the mesencephalon, particularly the boundary between prethalamus and thalamus (the zona limitans intrathalamica; [49], [50]). Here, the activation of ERK1/2 by the FGF8b bead was distributed symmetrical around it. In addition, this non-polarized ERK1/2 activity related to FGF8b signal was also detected in FGF8 hypomorphic mice (see Figure 4 and [45]) and in ONTCs experimental assays where both the anr and the IsO were ablated (see Figure 5 type 2 experiments). Importantly, when the IsO was ablated we could reverse the polarization effect of ERK1/2 activity, suggesting that in fact these FGF8-related morphogenetic centers are implicated in the differential ERK1/2 response. Interestingly, in these IsO ablation assays we did not detect any trace of FGF8 negative modulators gene expression on the remaining mesencephalon, raising the question whether other unknown rostral factors might also contribute to the reversed ERK activity. Viera et al., [67] using chick neural tube embryos and FGF8-beads implantation assays studied the molecular mechanisms by which *Pax2* gene expression pattern was restricted from diencephalic/mesencephalic boundary to the isthmic territory. 24 hours after FGF8b bead implantation in the caudal diencephalon, the authors showed a heterogeneous ectopic *Pax2* expression, which was consistently more intense on the caudal side of the FGF8b bead compared to the rostral side. These authors explained this phenomenon as a mechanism by which the putative signal that progressively restricted *Pax2* into the isthmic region could be of positive character (necessary to maintain *Pax2* expression). Indeed, this signal would probably come from the caudal part of the mesencephalon or the isthmus, needed for normal antero-posterior polarity of the epithelium and therefore would not be directly related to an FGF8 signal.

Ubiquitination by c-Cbl on the intracellular domain of the FGFR1 receptor leads to differential recycling of the receptor and modifies the duration of its signal [41], [68]. Based on the high affinity and sensitivity of this receptor to FGF8b signaling during neural development, we analyzed its expression profile in our secondary organizers ablation assays (Type 2 assays; Figure 5G; [51], [52]). In these experiments, the rather uniform expression of *FgfR1* in the mesencephalon was not affected during the time of the experiments (4hours; Figure 5H-I). Thus, our results suggest that instructions of FGF8 signal activity in mouse secondary organizers confer planar positional information from the IsO and from the anr by differentially di-phosphorylating ERK1/2 nearby neuroepithelial cells away from them without affecting the *FgfR1* gene expression (Figure 6K). Actually, this polarization coming from the two transversal secondary organizers seem to converge at the central diencephalic anlage (the zli) where no ERK differential polarizing activity occurs, leaving this brain area exempted from Fgf8-related secondary organizers influence activity before E9.5 stages (Figure 6K).

Then, what molecular mechanisms are behind this unbalanced activation of ERK1/2? Mutant mice have been used to understand the function of FGF8 negative feedback modulators in the mouse brain (i.e. *Mkp3, Sprouty1/2, Sef*). However, this powerful approach faces the difficulty of dissecting the function of each modulator because of their redundancy in FGF8 signal modulation [40], [69], [70], [71]. In this report, we used Brefeldin [53] to retain FGF8 molecules inside FGF8-producing cells at the IsO and anr and thus, eliminating the endogenous source of extracellular FGF8 protein along the neural tube for 4 hours. Under these deprivation conditions, the isthmic cells were the first affected cells in terms of ERK phosphorylation activity followed by the abutting territories (Figure 6 and 7). We still found traces of ERK1/2 activity outside this negative domain indicating a remanent FGF8 activity still ongoing. Importantly, *Mkp3* expression was concomitantly downregulated in the same domain where phosphorylated ERK1/2 was not immunodetected. On the contrary, expression of S*prouty1* and especially *Sprouty2* was maintained. Moreover, during BFA treatment FGF8-bead implantation on caudal mesencephalon maintained ERK1/2 polarized activation, indicating that *Mkp3* and probably *Sef* were not required in the specification of FGF8 differential positional planar induction activity in the mesencephalon. It has been proposed that SPROUTY 1/2 and SEF function synergistically to regulate *Gbx2* expression in the anterior hindbrain (a downstream target of FGF signaling; [70]). Suzuki-Hirano and collaborators [32] elegantly demonstrated that *Mkp3* was induced in chick neuroepithelial cells when a high level of ERK1/2 phosphorylation occurred. In agreement with the latter results, we were able to detect ectopic induction only of *Mkp3* after 3 hours of FGF8b soaked bead implantation in mouse ONTCs (Figure S1; [46]). On the other hand, in our BFA treatment assays *Mkp3* expression was the first modulator to be downregulated. Also, the same group demonstrated that *Sprouty2* was involved in the downregulation of ERK1/2 activity after its initial upregulation by FGF8 signal, and that this was required for proper mid-hindbrain differentiation. In our mouse ONTCs model system, *Sprouty2* may also be important for the correct early establishment of FGF8 positional information coming from the IsO by maintaining downregulated ERK1/2 phosphorylation levels. It is true that implantation of FGF8 soaked beads or ectopic gene expression by tissue electroporation may surpass the physiological levels of the protein. However, our results corroborated and provide a logical explanation of other previous works at which an ectopic source of the FGF8b protein either in caudal diencephalic and rostral mesencephalic territories (in terms of electroporation, grafted tissue and/or soaked beads), caused a mirror-like cerebellar tissue induction rostrally to the ectopic implanted source. Within our results we can conclude that those results were due to the first polarized ERK1/2 activity driven by the FGF8b signal [10], [72], [73]. Also the present work strongly suggests that the positional information given by FGF8 morphogen activity from IsO at E9.5 is coded already by the receptor response at the cytoplasmic level. This response is translated in distinct ERK1/2 phosphorylation states inside the neuroepithelial cells produced by the distinct levels and combinations of the FGF8 negative modulators. Actually, the decreasing gradients of FGF8 downstream regulators (mainly *Sprouty1/2*) at both sides of the IsO epithelium would maintain basic FGF8 intracellular activity to extend and to equilibrate the long-range distribution of active ERK1/2 along the A-P axis [32]. These results fit well with Meinhardt’s mathematical model for positional information signaling and establishment [74]. He proposed that the formation and maintenance of organizer regions would dependent of a short-ranging autocatalytic activator (the FGF8 in our model), which would catalyze in addition its long-ranging antagonist, the inhibitor (here, the SPROUTY family).

Finally, the immunodetection of FGF8 protein assays in embryos and ONTCs revealed staining domains at the ventricular side and at the basal lamina (see also [54]). Interestingly, under BFA treatment conditions, we unmasked that FGF8b protein was highly accumulated, in a vesicular-like manner, at the ventricular side of the neuroepithelial cells, which indicates that FGF8-expressing cells may secrete the morphogen to the lumen of the ventricle. However, as the ventricular surface area is very small, the apically localized vesicles may also be released to the basolateral side. Also, other recent reports claimed that FGF8-protein is highly concentrated at the basal lamina suggesting that FGFs may act through basal processes of neuronal progenitors to maintain their progenitor status [54]. In fact, mouse *Mkp3* is strongly expressed in mesenchyme compartment adjacent to the basal lamina at the isthmic region (see Figure 1 in [46]). In embryos, our monoclonal antibody immunodetection (See Figure S2 and material and methods) experiments showed positive immunolabeling of FGF8 protein at both ventricular zone and basal lamina with similar intensity. The same pattern was concomitantly observed in our ONTCs experimental model. In agreement with these observations, the high affinity FGF receptors related with the activation of FGF signaling pathways (*Fgfr1* and *2*) are predominantly expressed at the ventricular zone in E11.5 mouse embryos [75]. Furthermore, the resulting BFA treatment conditions in ONTCs revealed the lack of accumulating FGF8 positive staining at the basal lamina side. The release of the FGF8b protein from the ventricular side and its localization also on the basal lamina suggests a later transport of the protein from the apical to the pial side. Thus, alternative sorting and transcytosis mechanisms of FGF8b may occur inside the targets cells. Whatever the exact mechanism, our results further support association of FGF8 protein with basal lamina showing that establishment of the basal FGF8 gradient requires active exocytosis. Very recently and relevant published findings in chick claimed that FGF8b may also translocate into the nucleus, and this nuclear FGF8b could function as a transcriptional regulator to induce *Sprouty2* in the isthmus independently of ERK phosphorylation [76]. These new data in chick IsO together with our BFA assays where *Sprouty2* gene expression pattern was maintained in the absence of ERK1/2 activity provide new horizons of FGF8 function. In conclusion, FGF8 may exert distinct signal responses depending on its cellular localization. These differential planar instructions may allow the segregation of neurogenic and proliferation signaling mechanisms or alternatively facilitate diffusion of FGF8-related activity through the basal lamina during vertebrate neural tube patterning [77].

## Materials and Methods

### Organotypic Neural Tube Culture Explants Technique (ONTCs)

Timed pregnant mice were sacrificed by cervical dislocation and embryos were dissected in ice-cold phosphate-buffered saline (PBS; 0.1 M). The embryonic day (E) 0.5 was the noon of the day of the vaginal plug. The embryonic age was determined more precisely by counting the somites in which at E9.5 ranged between 21 and 29 somite pairs [78]. Anterior neural tube of E9.5 embryos was opened along the dorsal midline, placed on polycarbonate membranes (MilliCell PICMORG50), with the ventricular side facing up, and cultured in a 5% CO2, 100% humidity incubator at 37°C for up to 24 hours as previously described [42]. After experimental manipulation the explants were fixed in 4% paraformaldehyde in PBS between 2 and 4 hours. For the ablation experiments, the isthmic and anterior neural ridge regions of ONTCs were cut with the help of micro-scalpel blades (EagleLabs EG-4738) and cultivated on the same polycarbonate membranes, for 24 hours before bead implantation and/or chemical treatments (see below).

All animal manipulation and experimental procedures were performed accordingly to the directives of the Spanish and European Union governments (Council Directive 86/609/EEC) and approved by the Animal Experimentation Committee of the Institute of Neuroscience UMH-CSIC. Mice from ICR strain were used as wild type. The transgenic mouse strain Fgf8^neo/null^ (Fgf8 hypomorph mice in this paper; [45] were used as severe Fgf8 reduction level mice model [23], maintained on a C57BL/6 genetic background and generated as previously described by these last authors. En1^Cre/+^; Fgfr1^flox/flox^ were generated by crossing En1^Cre/+^; FgfR1^flox/+^ males with FgfR1^flox/flox^ females in outbred (129sv/ICR) background and genotyped as previously described by Trokovic et al., [50], [51].

### Implantation of FGF8b-soaked Beads

Heparin acrylic beads (Sigma-Aldrich H-5263) were rinsed in PBS and soaked in FGF8b solution (1 mg/ml; R&D) for 1 h at 4°C. FGF8b-soaked beads were rinsed three times in PBS and thereafter implanted in the neural tube explants cultures as previously described [79]. Control beads were incubated in PBS and implanted in the same manner.

### Bafilomycin A1 and Brefeldin A Treatments

Bafilomycin A1 (BAF; SigmaB1793) was used for blocking the lysosomal pathway and so, preventing FGF8 degradation after endocytosis [68]. ONTCs were incubated with BAF at a concentration of 1 µM in 0.04 mg/ml Dimethyl sulfoxide (DMSO; Sigma-Aldrich D8418; [47]), added to the culture medium (see protocol at [42]). The incubation time period was set at 2 hours, 37°C. Control explants were treated only with DMSO at same concentration and for same period of time.

Brefeldin A (BFA; Sigma B5936) was used for blocking release of exocytic vesicles content (including FGF8 secretion; [55]). Culture medium solution with the diluted chemical was used to treat the ONTCs, at a concentration of 25 µg/ml (Dahl et al., 2000), in 0.04 mg/ml DMSO. The incubation time period was set at 4 hours, 37°C for optimal desired effects. Control explants were treated with DMSO only as above mentioned.

### In Situ Hybridization (ISH) and Immunohistochemistry (IHC)

E9.5 whole-embryos were fixed in 4% PFA overnight at 4°C. Next day, samples were rinsed in PBT (PBS pH 7.4, with 0.1% Tween 20), dehydrated in an ascending ethanol series, and stored in 70% ethanol at 20°C before processing. Whole-mount ISH was performed according to Garda et al. 2001 protocol [77]. Digoxigenin-labeled RNA probes (DIG-11-UTP, Roche Diagnostics 11209256910) were detected by alkaline phosphatase-coupled anti-digoxigenin (Roche Diagnostics 11093274910), and incubation with BM-Purple substrate (Roche Diagnostics 1442074) as chromophore. After the colorimetric detection, embryos ONTCs were washed several times in PBT.

In the case of IHC procedure whole mount embryos where dissected in ice cold PBS and fixed in 4% PFA with phosphatase inhibitor tablets (Roche Diagnostics 04906837001) following companies protocol for 2–4 hours before starting procedure. In some cases and after ISH or immunostaining procedure, embryos or ONTCs were immersed in ascending sucrose to 30% concentration and then cut at 12 µm thick sections ONTCs in a cryostat at -26°C (Microm-ThermoFischer Scientific) for a cellular analysis.

Whole mounts embryos, ONTCs and cryostat tissue sections were rinsed 3 times in PBS 1× with 0,1% Triton (PBS-T) and then incubated with hydrogen peroxide (H_2_O_2_) at 3% for 30 minutes to inactivate the endogenous peroxidase activity. Then after 3 washes in PBS-T, they were blocked with goat serum at 5% and bovine serum albumin (BSA; Sigma-Aldrich A2153-50G) at 2% in PBS-T. Incubation of Rabbit anti-dpERK (1∶250; Cell Signaling Technologies #9101) was done overnight at room temperature (RT). For the immunodetection *in toto* of the di-phosphorylated form of ERK1/2, the primary antibody was incubated for three nights at 4°C. In the case of mouse anti-FGF8b (1∶250; R&D MAB323) for 2 nights at 4°C. Then, several washes in PBS-T were done before 1 hour incubation with Anti-rabbit or Anti-mouse biotinylated secondary antibodies at 1∶300 (Vector Laboratories BA-1000, BA-2020). Afterwards, Avidin-Biotin Complex was added at 1∶300 for 1 hour and washed in PBS-T (ABC kit; Vector Laboratories CA-94010). Colorimetric detection in embryos, ONTCs and tissue sections were incubated with 3,3′-Diaminobenzidine (DAB; Vector Laboratories SK-4100) and 0,003% H_2_O_2_. In some cases we used combined protocols of ISH and IHC within the same tissue. Finally for immunofluorescence detection of mouse monoclonal anti-FGF8b in cryostat sections an anti-mouse Alexa-Fluor-594; (1∶500: Molecular Probes A-11032) was used for 1 hour at RT. DAPI staining (1∶4000; Invitrogen #D1306) was used to visualize the nuclei of the cells. After all colorimetric detection, embryos ONTCs were washed several times in PBT. All images were photographed with Leica stereoscope (Leica MZ16FA) or an upright microscope (Leica DM6000B) for the cryostat sections, using a Leica DC500 camera or DCF350 camera for fluorescence images. All pictures were taken using Leica LAS AF software.

## Acknowledgments

We are grateful to Dr. Constantino Sotelo for insightful and helpful suggestions during preparation of the manuscript. We would like to thank specially Drs. Laura Lahti and Joan Galcerán for technical suggestions. We thank Dr. Gail Martin form kindly providing the mouse strain Fgf8^neo/null^ (Fgf8 hypomorph mice in this paper). We also thank Martineźs Laboratory Staff for technical support.

## Supporting Information

Figure S1 **Maintenance of molecular isthmic organizer signal activity in mouse organotypic tissue cultures (ONTCs).** Gene expression profile in mouse isthmic organizer by in situ hybridizations in mouse E9.5 ONTCs (A’-D’) in comparison to in toto mice of same age (A-D) after 6 hours of incubation. Mouse brain subdivisions at E9.5 ONTCs are described with the expression of *Meis2* in blue compared to *Fgf8* in red (A’) genes and one half of the explant. The transversal black dashed lines illustrate the boundaries depicted by the genes on the mouse brain tissue. B-D) FGF8 negative feedback modulators, *Sprouty2* (B), *Mkp3* (C), and S*ef* (D). Note the similarities of these genes with respect to that of *Fgf8* expression but the wider territory occupancy of their signals when compared to that of *Fgf8*, arguing indirectly the long range of FGF8 signal activity through the neuroepithelium from organizer centers.
(TIF)

Figure S2 **Expression pattern profile of** ***Fgf8*** **mRNA versus FGF8 protein in mouse E9.5 embryo.** An anti-FGF8b immunohistochemistry was made onto 12µm cryostat longitudinal sections to the isthmus (see drawing) to visualize the intracellular and extracellular FGF8b protein (see arrows for the expansion of the protein in C) and compared with the *Fgf8* mRNA domain (solid line in A,B). Note that the FGF8b protein can detected either at the ventricular side and at the pial side (see the white and black arrows sin D; see also [54]) and in forms of aggregates as vesicle-likes structures (small arrows in D).
(TIF)
