# Supplementary figures and images for: Fgf8-Related Secondary Organizers Exert Different Polarizing Planar Instructions along the Mouse Anterior Neural Tube
Source: PLoS One. 2012 Jul 6;7(7):e39977. doi: 10.1371/journal.pone.0039977 (PMC3391221; doi:10.1371/journal.pone.0039977)

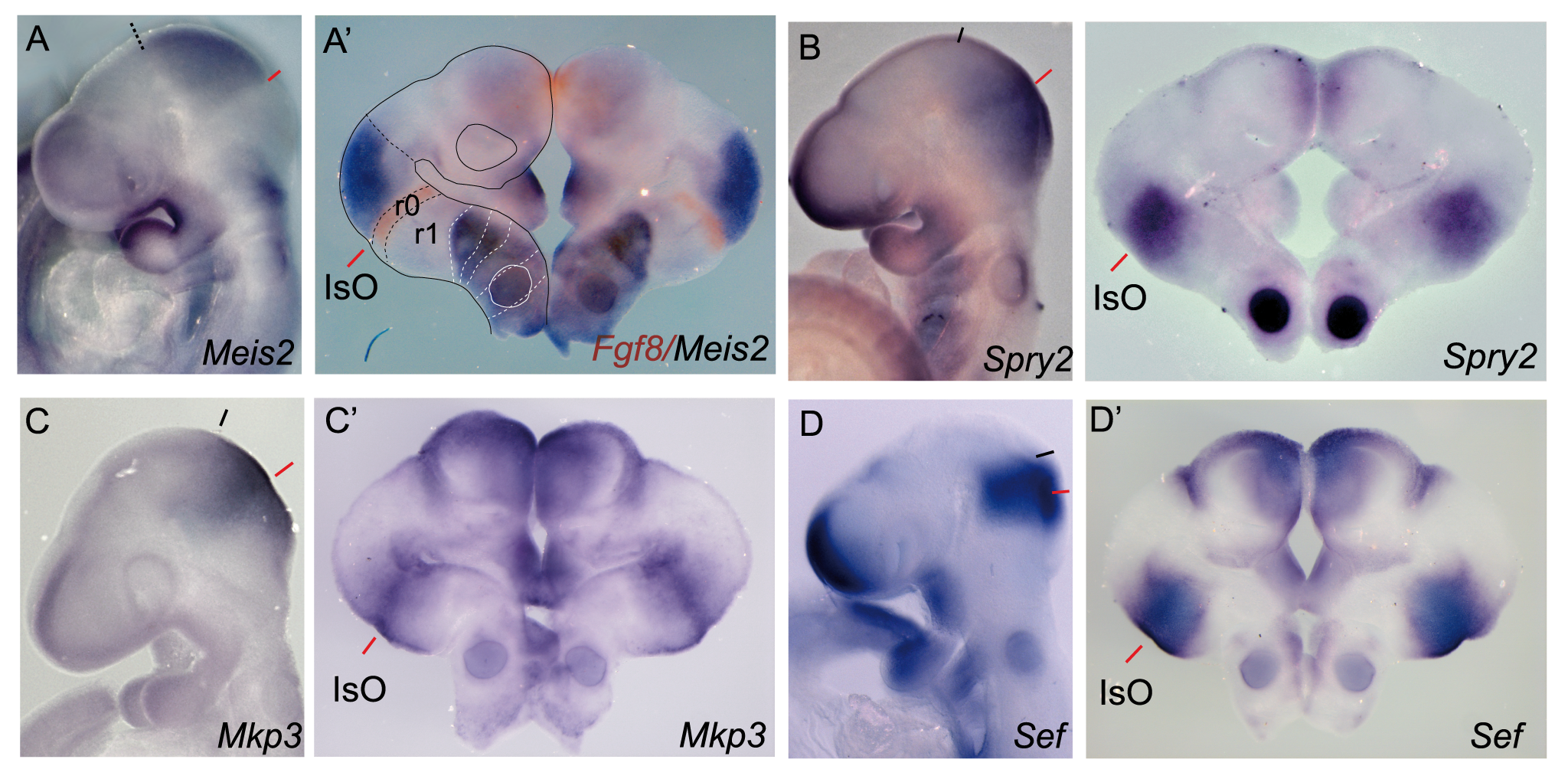

Supplement: Figure S1 — Maintenance of molecular isthmic organizer signal activity in mouse organotypic tissue cultures (ONTCs). Gene expression profile in mouse isthmic organizer by in situ hybridizations in mouse E9.5 ONTCs (A’-D’) in comparison to in toto mice of same age (A-D) after 6 hours of incubation. Mouse brain subdivisions at E9.5 ONTCs are described with the expression of Meis2 in blue compared to Fgf8 in red (A’) genes and one half of the explant. The transversal black dashed lines illustrate the boundaries depicted by the genes on the mouse brain tissue. B-D) FGF8 negative feedback modulators, Sprouty2 (B), Mkp3 (C), and Sef (D). Note the similarities of these genes with respect to that of Fgf8 expression but the wider territory occupancy of their signals when compared to that of Fgf8, arguing indirectly the long range of FGF8 signal activity through the neuroepithelium from organizer centers. (TIF) [file pone.0039977.s001.tif]

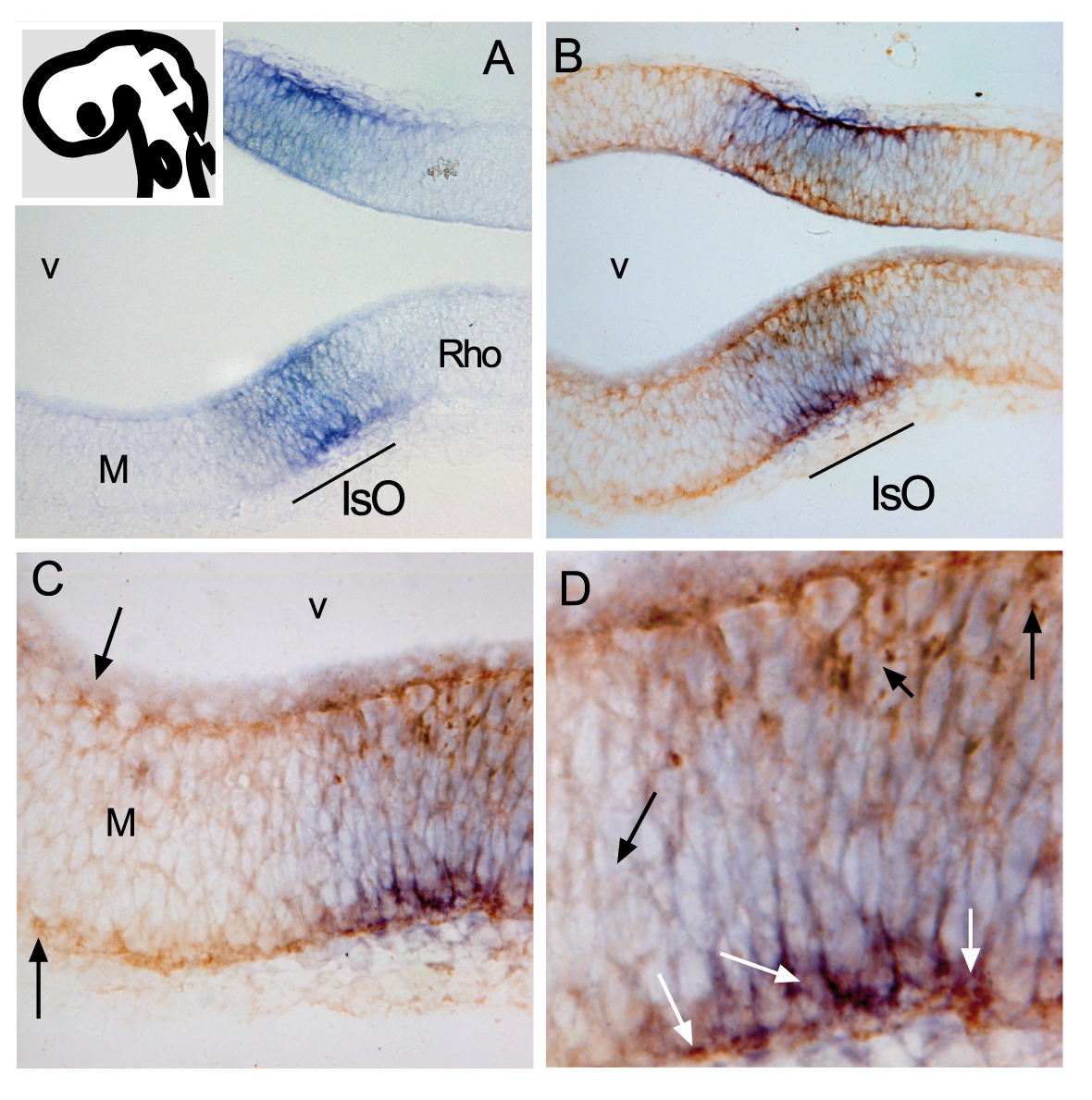

Supplement: Figure S2 — Expression pattern profile of Fgf8 mRNA versus FGF8 protein in mouse E9.5 embryo. An anti-FGF8b immunohistochemistry was made onto 12µm cryostat longitudinal sections to the isthmus (see drawing) to visualize the intracellular and extracellular FGF8b protein (see arrows for the expansion of the protein in C) and compared with the Fgf8 mRNA domain (solid line in A,B). Note that the FGF8b protein can detected either at the ventricular side and at the pial side (see the white and black arrows sin D; see also [54]) and in forms of aggregates as vesicle-likes structures (small arrows in D). (TIF) [file pone.0039977.s002.tif]
